# Unveiling Diagnostic Biomarkers in Autism: A Comparative Proteome Analysis of *CNTNAP2* Knockout Mice and Human ASD Patients

**DOI:** 10.3390/biom16030340

**Published:** 2026-02-24

**Authors:** Andrew Kim, Ara Cho, Jiyeon Kim, Leandro Val Sayson, Hyun Ju Lee, Jae Hoon Cheong, Hee Jin Kim, Bung Nyun Kim, Eugene C. Yi

**Affiliations:** 1 Department of Molecular Medicine and Biopharmaceutical Sciences, Graduate School of Convergence Science and Technology, Seoul National University, 103 Daehak-ro, Jongno-gu, Seoul 03080, Republic of Korea; kkim39@snu.ac.kr (A.K.); ara.cho@snu.ac.kr (A.C.); jy_kim@snu.ac.kr (J.K.); 2 Uimyung Research Institute for Neuroscience, Department of Pharmacy, Sahmyook University, 815 Hwarang-ro, Nowon-gu, Seoul 01795, Republic of Korea; lsayson@uwo.ca (L.V.S.); hjkim@syu.ac.kr (H.J.K.); 3 Department of Pediatrics, Hanyang University College of Medicine, Seoul 04763, Republic of Korea; blesslee77@hanyang.ac.kr; 4 Institute for New Drug Development, School of Pharmacy, Jeonbuk National University, 567 Baekje-daero, Deokjin-gu, Jeonju-si 54896, Republic of Korea; cheongjh@jbnu.ac.kr; 5 Department of Psychiatry and Behavioral Science, College of Medicine, Seoul National University, 101 Daehak-ro, Jongno-gu, Seoul 03080, Republic of Korea

**Keywords:** Autism Spectrum Disorder, proteomics, cross-species validation, machine learning, biomarkers

## Abstract

Autism Spectrum Disorder (ASD) is a biologically heterogeneous neurodevelopmental condition, presenting a major barrier to the identification of robust and translatable molecular biomarkers. Here, we employ a cross-species proteomic framework to identify conserved protein signatures associated with ASD. Quantitative proteomic profiling of brain and serum from *CNTNAP2* knockout mice, integrated with serum proteomes from individuals with ASD, revealed 132 proteins consistently dysregulated across species. Functional pathway analyses implicated coordinated alterations in lipid metabolism, synaptic signaling, and immune regulation. To prioritize diagnostically informative candidates, we applied machine learning-based feature selection and identified a minimal panel of ten proteins (COL1A1, ITIH4, CLU, NID1, C5, MASP1, PON1, PLTP, HSPA5, and FETUB) that robustly discriminated ASD from control samples. Gene ontology and KEGG pathway analyses highlighted enrichment of immune regulatory pathways, synaptic transmission, oxidative stress responses, and lipid metabolic processes, consistent with emerging models linking neuroimmune dysregulation and metabolic imbalance to ASD pathophysiology. An XGBClassifier trained on this biomarker panel achieved strong performance in independent test sets (AUC = 0.75). Together, these findings establish cross-species proteomic integration combined with machine learning as a powerful strategy for uncovering conserved, biologically grounded biomarkers in ASD, providing a framework for future validation and translational development.

## 1. Introduction

Autism Spectrum Disorder (ASD) is a heterogeneous neurodevelopmental condition characterized by deficits in social communication and interaction, along with restricted and repetitive behaviors, affecting approximately 1–2% of the global population [1]. According to current diagnostic frameworks, ASD symptomatology is organized into two core domains: impairments in social communication and interaction, and restricted, repetitive behaviors or interests [2]. The rising prevalence of ASD is widely attributed to evolving diagnostic criteria, improved screening practices, and increased public awareness.

Despite extensive research, the etiology of ASD remains complex and multifactorial, involving both genetic and environmental contributions [3]. Twin studies demonstrate high heritability estimates ranging from 64% to 91%, while prenatal and perinatal factors such as maternal infection, metabolic conditions, and inflammation have also been associated with increased risk [4,5,6]. Large-scale genomic studies, including those conducted by the Autism Sequencing Consortium, have identified mutations in over 100 genes associated with ASD, underscoring its profound genetic and phenotypic heterogeneity [7]. Clinically, this heterogeneity manifests as wide variability in cognitive, language, and social functioning among affected individuals [1].

At present, ASD diagnosis relies primarily on behavioral assessments and parent-reported questionnaires, such as the Modified Checklist for Autism in Toddlers, Revised with Follow-Up (M-CHAT-R/F) and the Social Communication Questionnaire (SCQ), followed by comprehensive clinical evaluations using instruments including ADOS-2 and CARS-2 [8,9,10]. Although well validated, these approaches are time-consuming, resource-intensive, and susceptible to confounding by symptom overlap with other neuropsychiatric conditions, delayed diagnosis, or compensatory behaviors that mask core ASD features, highlighting an urgent need for objective, biologically grounded biomarkers [11].

Multi-omics approaches have emerged as powerful strategies for elucidating the molecular basis of ASD. In particular, proteomic studies have implicated alterations in pathways related to synaptic signaling, immune regulation, and mitochondrial metabolism in ASD pathophysiology [12,13]. However, most proposed biomarkers remain in early stages of validation and are frequently derived from single-species or single-omics studies, limiting their robustness, reproducibility, and translational relevance.

Here, we address these limitations by employing a cross-species proteomic integration strategy that combines brain and serum proteomes from the *CNTNAP2*-knockout (KO) mouse model—a well-established genetic model of ASD—with serum proteomic data from individuals with ASD. *CNTNAP2* plays a critical role in cortical development and neural connectivity, and its disruption produces behavioral and neurobiological phenotypes highly relevant to ASD [14,15,16,17]. Through this integrative approach, we identify a set of shared orthologous proteins that are detectable across mouse and human datasets, based on one-to-one orthology and cross-species presence. Importantly, these proteins represent cross-species–shared molecular features defined by protein identity and detectability, rather than proteins exhibiting conserved or directionally concordant differential expression across species.

By combining this cross-species proteomic framework with machine learning–based analyses, we prioritize biologically informed biomarker candidates for ASD diagnosis and stratification. This strategy provides a scalable and conceptually transparent framework for future studies aimed at evaluating cross-species concordance in differential regulation, mechanistic relevance, and clinical translation.

## 2. Methods

### 2.1. Patient Cohorts and Sample Collection

Serum samples were obtained following ethical guidelines approved by the Institutional Review Board (IRB) of Seoul National University Hospital (Approval No: 2008-116-1150). Written informed consent was obtained from all participants or their legal guardians prior to sample collection. The study adhered to the principles of the Declaration of Helsinki.

For constructing the discovery cohort, 75 ASD patients and 36 age-matched neurotypical controls were used.

### 2.2. High-Abundant Protein Depletion in Serum Samples

Fourteen high-abundance serum proteins (albumin, IgG, IgA, IgM, IgD, α1-acid glycoprotein, fibrinogen, haptoglobin, α1-antitrypsin, α2-macroglobulin, transferrin, and apolipoprotein A1) were depleted using Select Top14 Abundant Protein Depletion Mini Spin Columns (Thermo Fisher Scientific, Waltham, MA, USA) according to the manufacturer’s instructions. For each sample, 500 µL of serum was processed for high-abundance protein depletion. Following depletion, total protein concentration was determined using a NanoDrop 2000 spectrophotometer (Thermo Fisher Scientific). Approximately 30 µg of total serum protein was subjected to enzymatic digestion, and 2 µg of the resulting peptides were injected for LC–MS/MS analysis.

### 2.3. Preparation of Serum Proteomic Samples

Depleted serum proteins were denatured in urea (Sigma-Aldrich, Burlington, MA, USA), reduced with dithiothreitol, and alkylated with iodoacetamide (both from Sigma-Aldrich). Proteins were then enzymatically digested using a trypsin/Lys-C mixture (Promega, Madison, WI, USA) to generate peptides. Resulting peptides were desalted using C18 StageTip columns, vacuum-dried, and stored at −80 °C until LC–MS/MS analysis.

### 2.4. LC-MS/MS Analysis of Patient Serum Samples

Peptides were reconstituted in solution A (0.1% Formic Acid in HPLC water) (Sigma-Aldrich) and 10 µL of the sample was injected into an Ultimate 3000 HPLC system (Thermo Fisher Scientific). Samples were first passed through a trap column (Acclaim PepMap™ 100, 75 µm × 2 cm, C18, 2 µm, 100 Å; Thermo Fisher Scientific) to remove contaminants, and then separated on an analytical column (PepMap™ RSLC C18, 2 µm × 50 cm, 100 Å; Thermo Fisher Scientific). Chromatographic separation was performed using a 165-min gradient at a flow rate of 0.3 µL/min, starting from 2–5% solvent B (0.1% FA in 98% Acetonitrile) over 5 min, increasing to 35% B at 138 min, 70% B at 148 min, and returning to 2% B at 165 min. Eluted peptides were analyzed on an Orbitrap Exploris™ 480 mass spectrometer (Thermo Fisher Scientific) equipped with a nano-electrospray ion source. The spray voltage was set to 1.8 kV and the ion transfer tube temperature to 275 °C. Full MS scans were acquired at a resolution of 120,000 across a mass range of 350–1650 m/z. Data-independent acquisition (DIA) was performed using a variable isolation window strategy, with subsequent fragmentation of all precursors within each window. RAW files were processed with DIA-NN software (version 1.8.1) [18] using default parameters. Protein identification was performed against a comprehensive pan-human spectral library encompassing proteins from multiple human organs and sample types [19]. Protein abundance information was extracted from the output file report.pg_matrix.tsv.

### 2.5. Mouse Serum Sample Preparation

*CNTNAP2* KO mice were generated by breeding heterozygous *CNTNAP2* mice (F0, +/−; Jackson Laboratory, Stock No. 017482) with C57BL/6J mice (Hanlim Laboratory Animals Co., Hwaseong, Korea), followed by intercrossing of heterozygous offspring (F1, +/−). Age-matched C57BL/6J mice were used as wild-type (WT) controls, consistent with the genetic background of the *CNTNAP2* KO line. Mice were housed six per cage under controlled environmental conditions (12 h light/12 h dark cycle; lights on from 07:00 to 19:00; 22 ± 2 °C) with ad libitum access to food and water. All experimental procedures were conducted with efforts to minimize animal stress and in accordance with institutional animal care guidelines.

Mouse serum samples were prepared for proteomic analysis by depleting high-abundance proteins using the Multiple Affinity Removal Column Mouse 3 (Agilent Technologies, Santa Clara, CA, USA), which selectively removes the most abundant serum proteins to enhance proteome coverage and dynamic range. Following depletion, proteins were subjected to in-solution digestion. Briefly, samples were reduced with dithiothreitol, alkylated with iodoacetamide, and digested with trypsin (Thermo Fisher Scientific). Resulting peptides were desalted using C18 StageTip columns, vacuum-dried, and stored at −80 °C until LC–MS/MS analysis.

### 2.6. Mouse Brain Sample Preparation

Mouse brain tissues were homogenized in lysis buffer containing 8 M urea, 50 mM Tris–HCl (pH 8.0), 75 mM NaCl, 1 mM EDTA, 1 mM EGTA, and a protease and phosphatase inhibitor cocktail (Roche, Switzerland). Homogenates were briefly sonicated to ensure complete cell lysis and centrifuged at 14,000× *g* for 10 min at 4 °C to remove insoluble debris. The resulting supernatants containing soluble proteins were collected, and protein concentrations were determined using a bicinchoninic acid (BCA) assay (Thermo Fisher Scientific).

For proteolytic digestion, proteins were processed using S-Trap columns (Protifi, Fairport, NY, USA) according to the manufacturer’s protocol. Briefly, samples were reduced with dithiothreitol, alkylated with iodoacetamide, and digested with trypsin. Peptides were subsequently eluted from the S-Trap columns, desalted using C18 StageTip columns, and stored at −80 °C until LC–MS/MS analysis.

### 2.7. MS Analysis of Mouse Samples

Tryptic peptides were reconstituted in 0.1% formic acid (FA) and loaded onto an analytical column (PepMap™, Thermo Fisher Scientific). Mass spectrometric analysis was performed using a Q Exactive hybrid quadrupole–Orbitrap mass spectrometer (Thermo Fisher Scientific) coupled to an Ultimate 3000 nanoLC system. Peptides were separated using a 95-min linear gradient from 5% to 35% solvent B (0.1% FA in 98% acetonitrile) at a flow rate of 300 nL/min. Full MS scans were acquired at a resolution of 70,000 over an m/z range of 400–1400, followed by data-dependent acquisition of the top 10 MS/MS scans at a resolution of 17,500 using a normalized collision energy of 27%. Dynamic exclusion was set to 30 s.

RAW mass spectrometry data were converted to mzXML format using PEAKS Studio and searched against the *Mus musculus* UniProt database. The precursor mass tolerance was set to 10 ppm and the fragment ion tolerance to 0.8 Da. Carbamidomethylation of cysteine residues was specified as a fixed modification, while oxidation of methionine was included as a variable modification. Trypsin was selected as the proteolytic enzyme, allowing up to two missed cleavages. Peptide and protein identifications were filtered to achieve a false discovery rate (FDR) of <1% using a target–decoy database search strategy.

Relative protein quantification was performed using the Power Law Global Error Model (PLGEM) implemented in R. PLGEM was applied to normalize datasets, identify statistically significant differentially expressed proteins (DEPs), and estimate expression changes based on *p*-values and signal-to-noise ratios. Functional enrichment and pathway analyses of DEPs were conducted using Gene Ontology annotations through STRING [20].

### 2.8. Cross-Species Comparison

A cross-species comparative analysis was performed between *CNTNAP2* KO mice and human ASD cohorts to identify cross-species shared orthologous protein features. Proteomic datasets obtained from mouse serum, mouse brain, and human serum were mapped to their corresponding mouse–human orthologs using the g:Profiler platform [21] in conjunction with the UniProt database. Only proteins with unambiguous one-to-one orthologous relationships were retained for downstream analyses. Protein sets detected across datasets from both species were subsequently integrated to define a shared orthologous proteome, based on protein identity and cross-species presence rather than conserved or directionally concordant differential expression. This orthologous protein set was then subjected to functional annotation and network-based analyses to assess enrichment of biological pathways and molecular processes relevant to ASD.

### 2.9. Development of Machine Learning Models

Protein abundance features containing missing values were excluded from subsequent analyses. The remaining data were normalized using a robust scaler, which centers features by the median and scales them according to the interquartile range, thereby minimizing the influence of outliers. Feature selection was then performed using an Extra Trees Classifier to identify proteins most informative for distinguishing ASD samples from controls. To avoid data leakage and ensure unbiased model evaluation, feature selection was conducted exclusively on the training dataset, with the selected features subsequently applied to the test dataset for model assessment.

### 2.10. Cross-Validation and Model Selection

The dataset was randomly partitioned into training and test sets at an 80:20 ratio. To preserve class balance between ASD and control samples, stratified k-fold cross-validation (k = 5) was applied within the training dataset, maintaining consistent class proportions across folds. Multiple machine learning algorithms, including AdaBoost, logistic regression, random forest, XGBClassifier, decision tree, k-nearest neighbors (KNN), and linear support vector classification (Linear SVC), were evaluated using this fivefold cross-validation scheme for hyperparameter optimization and comparative performance assessment. The final model was selected based on cross-validation results and subsequently evaluated on the held-out test set. Model performance was quantified using accuracy, sensitivity, specificity, precision, recall, F1 score, and the area under the receiver operating characteristic (AUROC) curve to ensure comprehensive assessment of classification robustness. Unless otherwise stated, all reported cross-validation results refer to the same stratified fivefold cross-validation procedure applied within the training dataset.

## 3. Results

### 3.1. Proteome Analysis of CNTNAP2 KO Mice and Human ASD Patients

To characterize proteomic alterations associated with *CNTNAP2* deficiency and ASD, we performed quantitative proteomic profiling of serum and brain tissue from *CNTNAP2* knockout (KO) mice and serum from human ASD patients. In mouse serum, 1706 proteins were quantified, of which 589 were identified as differentially expressed relative to wild-type controls (*p* < 0.05; Supplementary Table S1).

In mouse brain tissue, a total of 8915 proteins were quantified, with 495 proteins showing significant differential expression compared with controls (*p* < 0.05; Supplementary Table S2).

In human ASD serum samples, 242 proteins were quantified, and 132 proteins were differentially expressed relative to neurotypical controls (*p* < 0.05; Supplementary Table S3).

**Figure 1:**
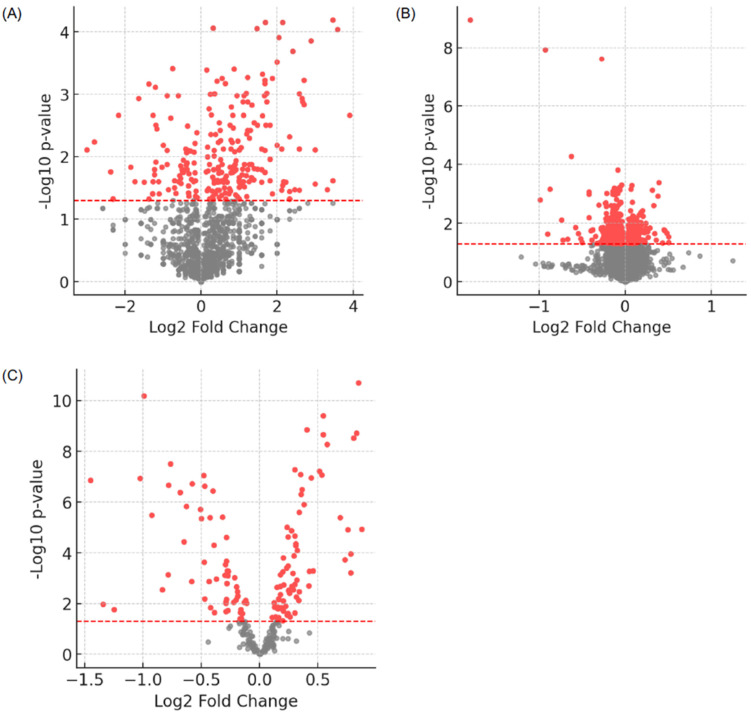
Differential proteomic profiles in *CNTNAP2* knockout mice and ASD patients. Volcano plots illustrating differentially expressed proteins (DEPs) in (**A**) serum from *CNTNAP2* knockout (KO) mice, (**B**) brain tissue from *CNTNAP2* KO mice, and (**C**) serum from ASD patients. The *x*-axis represents the log_2_ fold change in protein abundance, and the *y*-axis represents the −log_10_ *p* value. Proteins meeting the statistical significance threshold (*p* < 0.05) are highlighted in red.

The distribution of differentially expressed proteins (DEPs) in each dataset was visualized using volcano plots (Figure 1). In these plots, log_2_ fold change is displayed on the *x*-axis and –log_10_ *p*-value on the *y*-axis. Proteins with increased abundance are shown in red, whereas proteins with decreased abundance are shown in grey.

### 3.2. Cross-Species Comparative Analysis

To enable cross-species comparison between *CNTNAP2* KO mice and human ASD patients and to examine overlap between central and peripheral proteomes, complete proteomic datasets derived from mouse brain tissue, mouse serum, and human ASD serum were integrated. Rather than restricting the analysis to differentially expressed proteins, all quantified proteins from each dataset were included to maximize coverage of cross-species shared molecular features.

**Figure 2:**
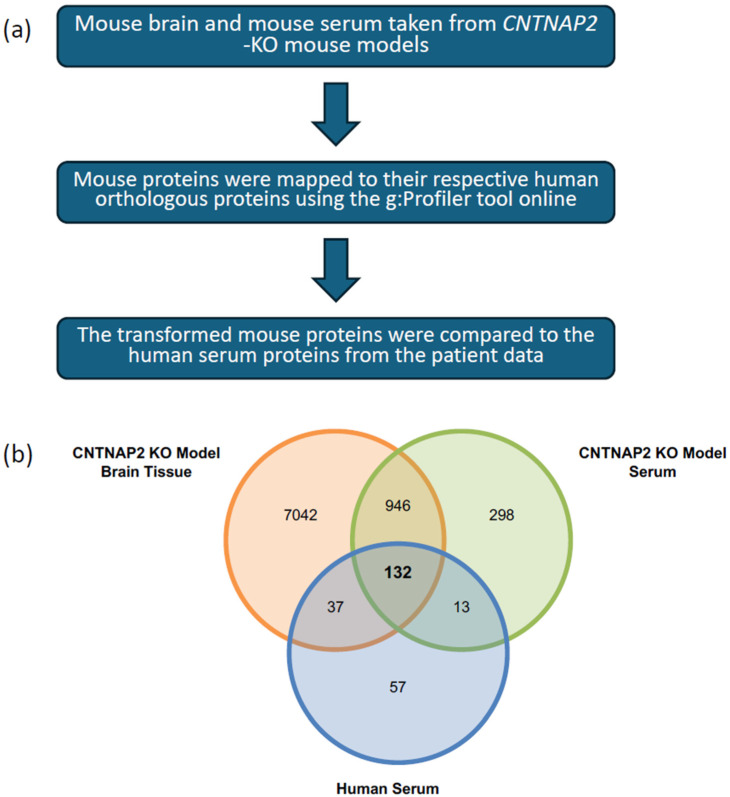
Cross-species protein mapping and identification of conserved ASD-associated proteins. (**a**) Schematic overview of the cross-species mapping workflow used to align proteins identified in *CNTNAP2* knockout (KO) mouse serum and brain proteomes to their corresponding human orthologs using the g:Profiler and UniProt databases. Only one-to-one orthologous protein pairs were retained for downstream analysis. (**b**) Venn diagram illustrating the overlap among proteomic datasets from *CNTNAP2* KO mouse serum, *CNTNAP2* KO mouse brain, and human ASD patient serum. A total of 132 proteins were commonly detected across species, representing conserved molecular features associated with ASD.

Mouse proteins were mapped to their human counterparts using g:Profiler in combination with the UniProt database. Only proteins with unambiguous one-to-one orthologous relationships were retained for downstream analyses. The overall workflow for ortholog mapping and dataset integration is summarized in Figure 2a.

Overlap among the three proteomic datasets was visualized using a Venn diagram (Figure 2b). A total of 1018 proteins were detected in both mouse brain tissue and mouse serum. Cross-species comparison identified 132 proteins that were commonly detected in mouse brain, mouse serum, and human ASD serum, representing a shared orthologous protein set defined by protein identity and cross-species presence (Supplementary Table S4).

**Figure 3:**
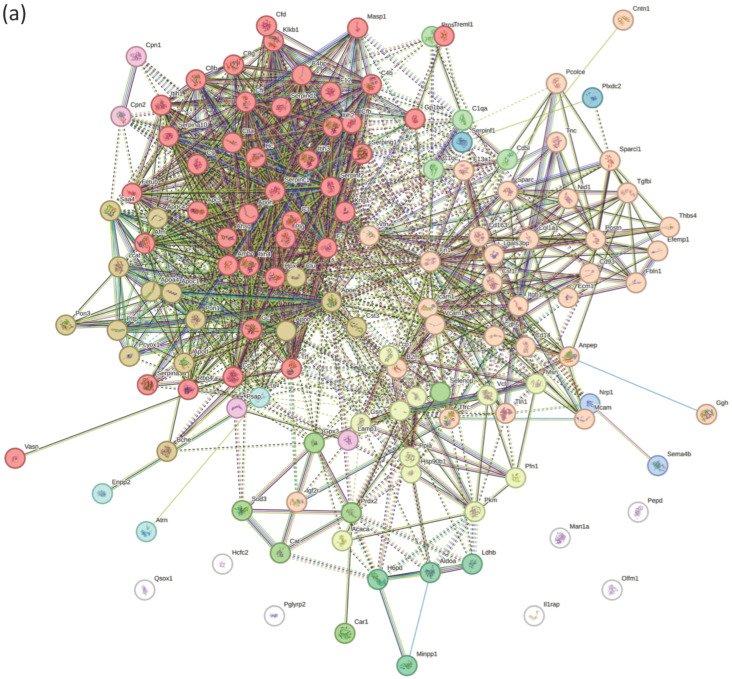

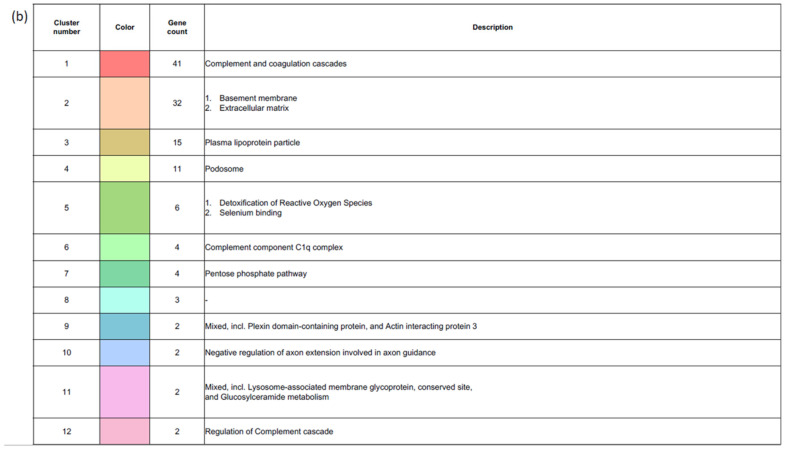
Protein–protein interaction (PPI) networks of conserved ASD-associated proteins in mouse. (**a**) PPI network constructed from conserved proteins identified in the *CNTNAP2* knockout (KO) mouse dataset. Nodes represent individual proteins, and edges indicate predicted functional associations. (**b**) Node colors denote enriched biological processes as indicated in the legend.

**Figure 4:**
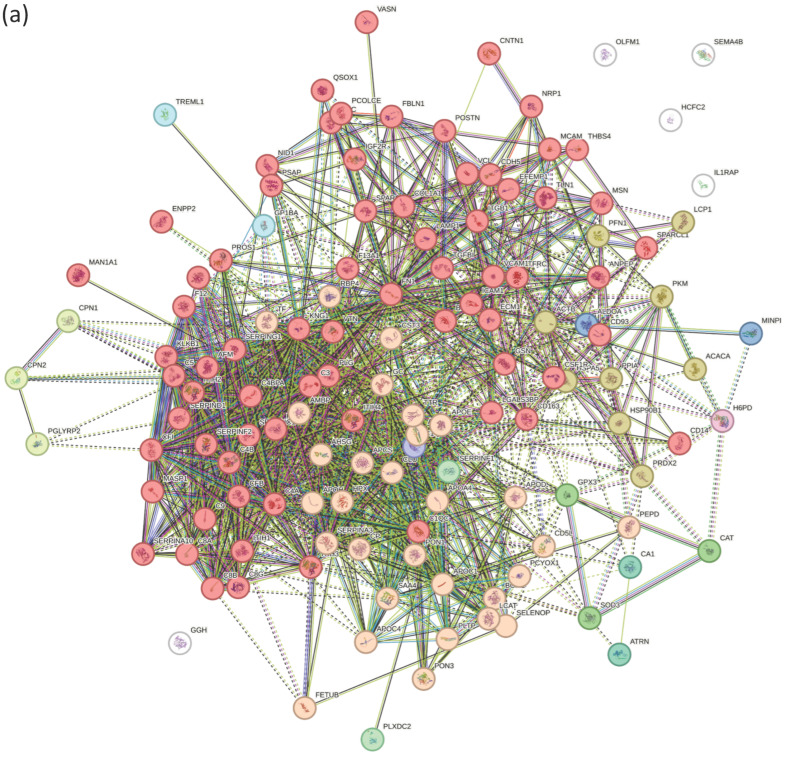

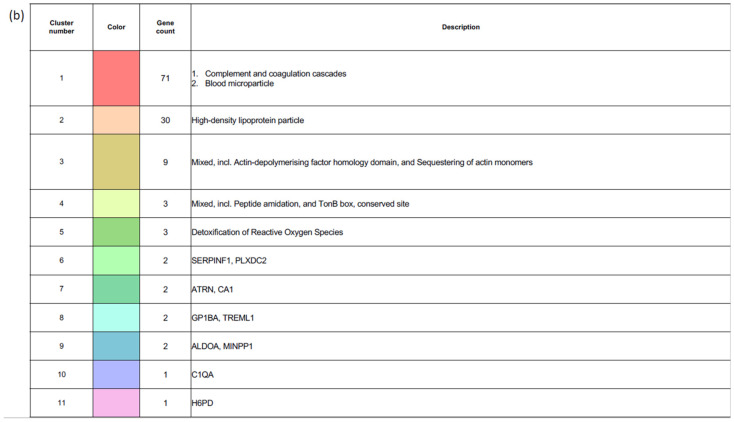
Protein–protein interaction (PPI) networks of conserved ASD-associated proteins in human datasets. (**a**) PPI network constructed from conserved proteins identified in the human ASD patient serum dataset. Nodes represent individual proteins, and edges indicate predicted functional associations. (**b**) Node colors denote enriched biological processes as indicated in the legend.

To further characterize functional relationships among the shared orthologous proteins, protein–protein interaction (PPI) network analyses were performed separately for the mouse and human datasets using STRING. The resulting interaction networks are shown in Figure 3 (mouse) and Figure 4 (human). Network clustering analysis revealed multiple groups of interacting proteins annotated to functional categories including complement and coagulation cascades, plasma lipoprotein particle metabolism, and oxidative stress–related processes [22,23].

### 3.3. Biomarker Panel Identification Using Machine Learning

**Figure 5:**
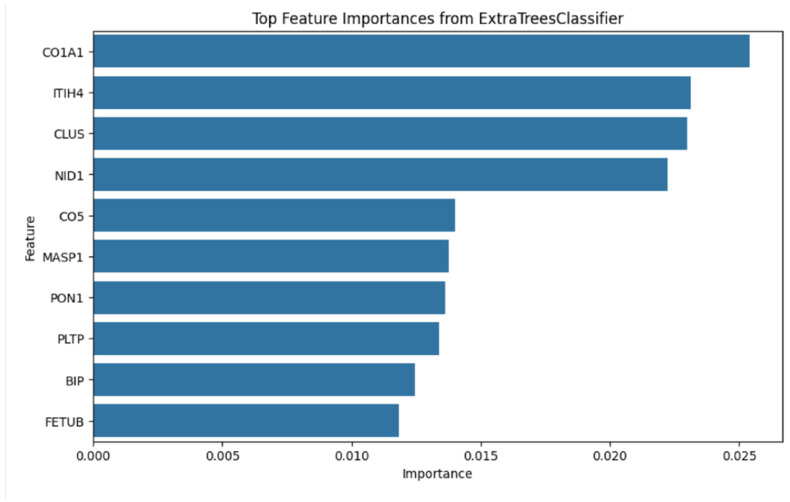
Identification of top candidate ASD biomarkers using machine learning. The top 10 protein features selected by the Extra Trees classifier are shown. Candidate features were drawn from the 132 proteins conserved across *CNTNAP2* knockout (KO) mouse and human ASD proteomes, as well as additional proteins identified through protein–protein interaction (PPI) network analysis. Features are ranked according to their relative importance scores, reflecting their contribution to distinguishing ASD samples from controls.

To identify protein features capable of distinguishing ASD samples from controls, we applied a supervised machine learning framework. Candidate features were derived from two sources: (i) the 132 proteins conserved across *CNTNAP2* KO mouse and human ASD proteomes, and (ii) additional proteins identified through protein–protein interaction (PPI) network analysis. Feature selection was performed using an Extra Trees Classifier, which ranks variables based on their relative contribution to classification performance as measured by feature importance scores derived from impurity reduction across the ensemble. The ten highest-ranking proteins were COL1A1, ITIH4, CLU, NID1, C5, MASP1, PON1, PLTP, HSPA5, and FETUB (Table 1). The ranking of these features based on their importance score is visualized in Figure 5.

To evaluate classification performance, multiple machine learning algorithms were trained and tested, including AdaBoost, Logistic Regression, Random Forest, XGBoost, Decision Tree, K-Nearest Neighbors, and Linear Support Vector Classifier. Model performance was assessed using accuracy, sensitivity, specificity, and area under the receiver operating characteristic curve (AUROC) on independent training and test datasets (Table 2). Among the evaluated models, the XGBClassifier showed the highest performance on the test set, with an accuracy of 0.78, sensitivity of 0.67, specificity of 0.82, and an AUROC of 0.82, and was therefore selected for subsequent analyses.

**Figure 6:**
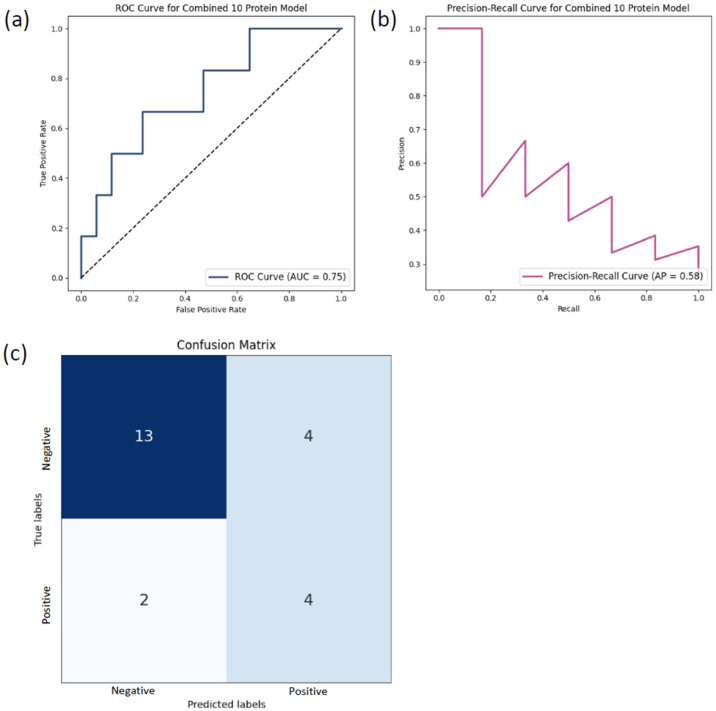
Performance evaluation of the XGBClassifier model. (**a**) Receiver operating characteristic (ROC) curve of the combined 10-feature model, showing an area under the curve (AUC) of 0.75, indicating robust discrimination between ASD and control samples. (**b**) Precision–recall curve of the same model, with a precision–recall value of 0.58, reflecting model performance under class imbalance conditions. (**c**) Confusion matrix summarizing the classification results of the XGBClassifier model. ASD samples are labeled as responsive (R) and controls as non-responsive (NR). The matrix depicts true positives (TP), true negatives (TN), false positives (FP), and false negatives (FN), providing a detailed assessment of model accuracy and highlighting its potential utility for ASD diagnostic applications.

The evaluation results of the XGBClassifier were visualized in Figure 6 using the ROC curve, which showed an AUC score of 0.75 and the precision-recall curve, which showed a precision-recall of 0.58, which shows high sensitivity and high precision of the XGBClassifier. Furthermore, the confusion matrix in Figure 6c showed high true positive rates and low false negative rates, showing the predictive accuracy of the XGBClassifier on the test set. These results reflect the performance of the selected features within the applied internal fivefold cross-validation framework and the held-out test dataset.

### 3.4. Differential Expression of Biomarkers in Serum of ASD Patients

**Figure 7:**
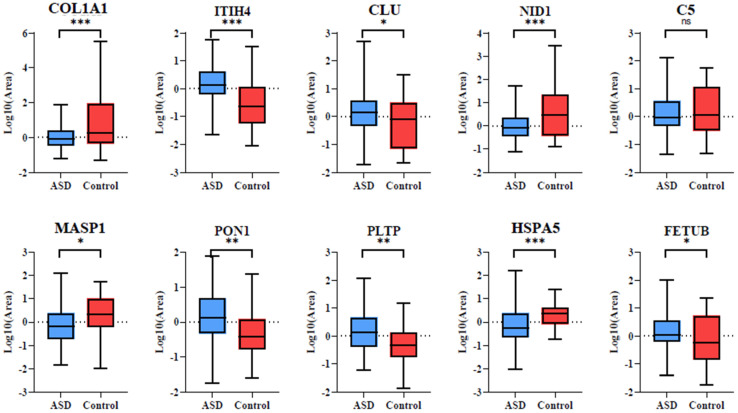
Relative quantitative abundance of the 10 selected protein biomarkers in control and ASD serum samples. Bar plots depict normalized protein expression levels, demonstrating significant differential expression between groups (Two-tailed *t*-test, * for *p* < 0.05, ** for *p* < 0.01, *** for *p* < 0.001, ns non-significant).

To further confirm whether these proteins could function as biomarkers in patient serum, the relative mass quantitative values of the biomarker panels were compared for control serum and serum from ASD patients. As shown in Figure 7, all proteins except complement component C5 showed significant differential expression between the control serum and the serum of ASD patients, which further strengthens our results and highlights the potential of this biomarker panel to be used in the diagnosis of ASD.

### 3.5. Pathway and Network Analysis

To examine the functional characteristics of the ten selected biomarker candidates (COL1A1, ITIH4, CLU, NID1, C5, MASP1, PON1, PLTP, HSPA5, and FETUB), pathway enrichment and network analyses were performed. Gene Ontology (GO) enrichment and KEGG pathway analyses were conducted across the biological process (BP), molecular function (MF), and cellular component (CC) categories.

**Figure 8:**
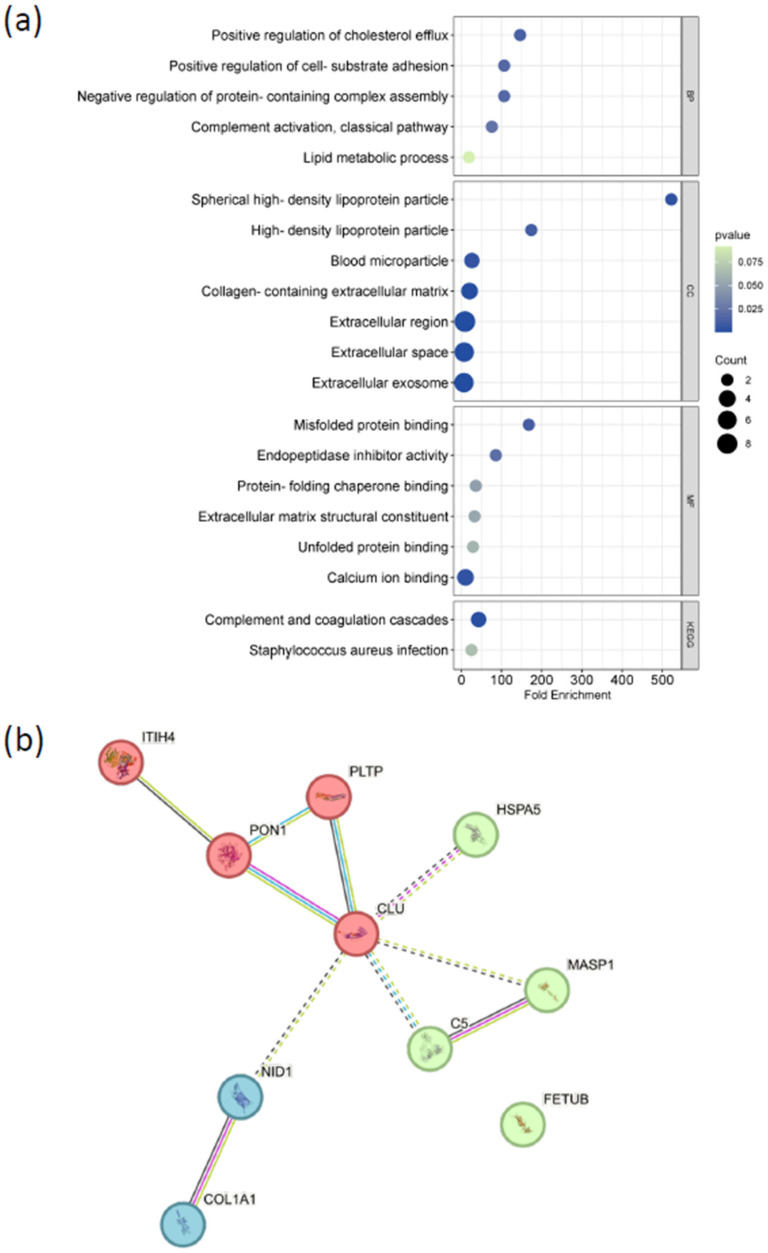
Functional enrichment and protein–protein interaction network analysis of the selected ASD biomarker panel. (**a**) Bubble plot summarizing Gene Ontology (GO) enrichment (biological processes [BP], cellular components [CC], molecular functions [MF]) and KEGG pathway analyses for the 10 candidate biomarkers. Bubble size corresponds to the number of proteins associated with each term, color indicates statistical significance (*p*-value), and fold enrichment reflects the degree of overrepresentation. Enriched pathways include synaptic signaling, immune response, cellular stress response, and metabolic regulation. (**b**) Protein–protein interaction (PPI) network of the selected biomarkers (COL1A1, ITIH4, CLU, NID1, C5, MASP1, PON1, PLTP, HSPA5, and FETUB) generated using the STRING database. Nodes represent individual proteins and edges indicate predicted functional associations. Distinct interaction modules are observed, with CLU acting as a central hub linking multiple functional clusters.

As shown in Figure 8a, the biomarker set was enriched for terms related to synaptic signaling, immune-related processes, cellular stress responses, and metabolic regulation. The complete list of significantly enriched GO terms and KEGG pathways, along with associated enrichment statistics, is provided in Supplementary Table S5.

Protein–protein interaction (PPI) network analysis was performed using the STRING database to evaluate interaction relationships among the ten proteins. The resulting network is shown in Figure 8b and reveals multiple interaction groupings. These included a lipid metabolism-associated cluster, an extracellular matrix-related cluster containing COL1A1 and NID1, and an immune-related cluster in which MASP1 appeared as a single-node interaction. CLU displayed multiple connections within the network and showed the highest degree of connectivity among the candidate proteins.

Together, these analyses characterize the functional annotations and interaction patterns associated with the selected biomarker candidates, providing an overview of their distribution across molecular pathways and biological processes relevant to ASD.

## 4. Discussion

In this study, we integrated proteomic data from *CNTNAP2* knockout (KO) mice and human ASD patients to identify a cross-species shared orthologous protein set comprising 132 proteins detectable across mouse brain, mouse serum, and human ASD serum. This protein set reflects shared molecular features defined by protein identity and cross-species presence, rather than directionally conserved differential expression. By applying a cross-species comparative proteomics strategy spanning central and peripheral compartments, we extend beyond single-species or single-tissue analyses and establish a framework for prioritizing candidate molecular features with potential relevance to ASD biology.

Unsupervised clustering analysis demonstrated that these shared orthologous proteins form functionally coherent groups, with prominent representation of high-density lipoprotein (HDL) particle–associated proteins. This observation highlights the utility of cross-species integration for identifying molecular pathways recurrently represented across experimental models and human disease contexts, even in the absence of strict concordance in differential regulation. However, given the well-recognized clinical and biological heterogeneity of ASD, the degree to which these shared molecular features generalize across distinct ASD subtypes remains an open question.

Building on this shared orthologous protein landscape, machine learning–based feature selection identified a panel of ten proteins—COL1A1, ITIH4, CLU, NID1, C5, MASP1, PON1, PLTP, HSPA5, and FETUB—that discriminated ASD from control samples within the analyzed dataset. Importantly, these results were derived from internal cross-validation and a single train/test split using a clinically heterogeneous and unstratified ASD cohort, and should therefore be interpreted as preliminary and hypothesis-generating rather than indicative of broad diagnostic applicability. The absence of stratification by clinical subtype, symptom severity, sex, or comorbidities limits assessment of how ASD heterogeneity may influence both proteomic signatures and machine learning performance. Consequently, the identified biomarker panel should be viewed as representing molecular features present in a subset or aggregate of ASD cases, rather than a universally applicable diagnostic signature.

With the exception of C5, all candidate proteins were significantly differentially expressed in ASD serum, supporting their potential relevance as circulating biomarker candidates within the studied cohort. Notably, these proteins span multiple functional categories rather than converging on a single biological pathway, a pattern consistent with the molecular heterogeneity that characterizes ASD. This diversity suggests that different combinations of molecular perturbations may underlie distinct ASD phenotypes, and that future biomarker efforts may benefit from stratified or subtype-specific modeling approaches rather than a single global classifier.

Pathway enrichment and network analyses provided biological context for the identified candidate proteins. Gene Ontology and KEGG analyses highlighted processes related to synaptic signaling, immune regulation, oxidative stress responses, and metabolic pathways, all of which have been repeatedly implicated in ASD pathophysiology. Protein–protein interaction network analysis further organized the candidates into functional modules, including a lipid metabolism–associated cluster, an extracellular matrix–related cluster comprising COL1A1 and NID1, and an immune-associated node centered on MASP1.

Notably, proteins involved in lipid metabolism and oxidative stress were prominently represented. The brain is highly enriched in lipids, and cholesterol synthesized predominantly by astrocytes is transported to neurons via lipoprotein particles to support axonal integrity and synaptic connectivity. Disruptions in lipid and cholesterol metabolism have been linked to oxidative stress, cytotoxicity, and impaired neuronal function [24,25,26]. Proteins such as PON1, identified in this study, exert neuroprotective effects by mitigating cytokine- and microglia-derived reactive oxygen species and have been implicated in neurodegenerative disorders including Parkinson’s disease, Alzheimer’s disease, and amyotrophic lateral sclerosis [27,28]. These observations are consistent with prior reports of altered omega-3 and omega-6 polyunsaturated fatty acid profiles in ASD.

Within the network, CLU emerged as a highly connected hub, consistent with its established roles in lipid transport, complement regulation, and cellular stress responses [29,30,31]. This central positioning suggests a potential integrative role linking metabolic and immune pathways in ASD, rather than specificity to a single molecular subtype. Nevertheless, whether lipid-associated proteomic alterations represent shared core mechanisms or subtype-specific features of ASD will require validation in larger, phenotypically stratified cohorts.

Among the implicated pathways, immune dysregulation, particularly involving the complement system, has received increasing attention in neurodevelopmental disorders [32]. Complement signaling plays essential roles in neural progenitor proliferation, differentiation, migration, and synaptic pruning, and its dysregulation has been linked to ASD as well as other central nervous system disorders, including schizophrenia and Alzheimer’s disease [33]. Complement-related proteins such as C5 and ITIH family members have been associated with neuroinflammatory processes in ASD and related conditions [34,35], while ITIH proteins have also been implicated in inflammatory responses in bacterial infection, ischemic stroke, and Parkinson’s disease [36,37,38].These findings support the relevance of neuroimmune mechanisms in ASD, while also underscoring the likelihood that immune-related proteomic signatures vary across individuals and clinical subgroups.

## 5. Conclusions

Compared with earlier ASD biomarker studies that have focused primarily on genetic variation, immune markers, or oxidative stress in isolation, this work offers a complementary perspective by emphasizing cross-species shared orthologous protein features identified at the proteome level. By integrating cross-species proteomics with machine learning and network-based analyses, we prioritize candidate molecular features that may be relevant to ASD-associated biological processes, helping to connect molecular observations from animal models with those observed in human ASD samples.

Several limitations should be acknowledged. The modest cohort size limits statistical power. In future studies, an independent validation cohort will be needed to further validate our results. Furthermore, the reliance on a single ASD mouse model constrains coverage of the full biological and clinical heterogeneity of ASD. In addition, the machine learning analyses were based on internal cross-validation and a single train/test split, without independent external validation. Accordingly, the identified biomarker panel and associated performance metrics should be interpreted as exploratory and hypothesis-generating. Differential expression and pathway enrichment analyses were also performed using nominal significance thresholds without formal multiple-testing correction, further underscoring the need for cautious interpretation. Future studies incorporating larger, independent, and phenotypically stratified patient cohorts, additional ASD-relevant animal models, and complementary multi-omics approaches will be essential to evaluate generalizability, robustness, and potential clinical utility.

In summary, this study demonstrates that cross-species proteomic integration, coupled with machine learning and network-based analyses, can serve as a useful discovery framework for identifying candidate molecular features associated with ASD. While substantial validation remains necessary, the observed associations suggest that alterations in immune regulation, lipid metabolism, oxidative stress responses, and extracellular matrix organization may contribute to ASD-related molecular phenotypes. This integrative strategy provides a scalable foundation for future biomarker studies aimed at advancing biological understanding and supporting the development of rigorously validated molecular signatures in ASD.

## Figures and Tables

**Table 1:** Top 10 biomarkers and their importance scores. Top 10 candidate biomarkers ranked by feature importance as determined by the Extra Trees Classifier.

	Feature	Importance
1	COL1A1	0.0254
2	ITIH4	0.0231
3	CLU	0.0230
4	NID1	0.0222
5	C5	0.0140
6	MASP1	0.0138
7	PON1	0.0136
8	PLTP	0.0134
9	HSPA5	0.0124
10	FETUB	0.0118

**Table 2:** Performance metrics for machine learning models. A table detailing the accuracy, sensitivity, specificity, and AUROC, assessed in both training and independent test datasets.

Set	Algorithms	Accuracy	Sensitivity	Specificity	AUROC
Training set	AdaBoostClassifier	0.85	0.57	1.00	0.95
	LogisticRegression	0.81	0.67	0.88	0.85
	RandomForestClassifier	1.00	1.00	1.00	1.00
	XGBClassifier	1.00	1.00	1.00	1.00
	DecisionTreeClassifier	0.97	0.90	1.00	0.98
	KNeighborsClassifier	0.75	0.27	1.00	0.88
	LinearSVC	0.81	0.50	0.97	0.85
Test set	AdaBoostClassifier	0.74	0.33	0.88	0.86
	LogisticRegression	0.74	0.67	0.76	0.75
	RandomForestClassifier	0.78	0.50	0.88	0.82
	XGBClassifier	0.78	0.67	0.82	0.82
	DecisionTreeClassifier	0.65	0.33	0.76	0.44
	KNeighborsClassifier	0.83	0.33	1.00	0.84
	LinearSVC	0.74	0.33	0.88	0.75

## Data Availability

The proteomic raw data supporting the findings of this study are available in the PRIDE repository under the accession number PXD060810. The dataset can be accessed at https://www.ebi.ac.uk/pride.

## Supplementary Materials

The following supporting information can be downloaded at: https://www.mdpi.com/article/10.3390/biom16030340/s1, Table S1: Differentially expressed proteins identified in serum from CNTNAP2 knockout mice; Table S2: Differentially expressed proteins identified in brain tissue from CNTNAP2 knockout mice; Table S3: Differentially expressed proteins identified in serum from human ASD patients; Table S4: Cross-species shared orthologous proteins identified in mouse (CNTNAP2 KO) and human ASD proteomes; Table S5: List of significantly enriched Gene Ontology (GO) terms and KEGG pathways with corresponding enrichment statistics.
